# New insight into the role of extracellular vesicles in kidney disease

**DOI:** 10.1111/jcmm.14101

**Published:** 2018-12-25

**Authors:** Lin‐Li Lv, Ye Feng, Tao‐Tao Tang, Bi‐Cheng Liu

**Affiliations:** ^1^ Zhongda Hospital, Institute of Nephrology, Southeast University School of Medicine Nanjing China

**Keywords:** biomarker, cell communication, exosome, extracellular vesicles, kidney disease

## Abstract

Extracellular vesicles (EVs) are released to maintain cellular homeostasis as well as to mediate cell communication by spreading protective or injury signals to neighbour or remote cells. In kidney, increasing evidence support that EVs are signalling vesicles for different segments of tubules, intra‐glomerular, glomerular‐tubule and tubule‐interstitial communication. EVs released by kidney resident and infiltrating cells can be isolated from urine and were found to be promising biomarkers for kidney disease, reflecting deterioration of renal function and histological change. We have here summarized the recent progress about the functional role of EVs in kidney disease as well as challenges and future directions involved.

## 1 GENERATION AND PROPERTIES OF EXTRACELLUAR VESICLE (EV)

Extracellular vesicles are membrane structures released into extracellular space by a variety of cells. Currently, EVs are classified into three categories based on their biogenesis. Apoptotic bodies, with a diameter range from 200 nm to 5 μm, are shed from the plasma membrane of cells undergoing programmed cell death. Microvesicles (MVs) are shed from the plasma membrane of viable cells with size of 100‐800 nm. Exosomes are 30‐150 nm in size and are released into the extracellular space when multivesicular bodies (MVBs) fuse with the plasma membrane.^1^

Extracellular vesicles are secreted by most cell types under both physiological and pathological conditions. However, when cells are exposed to stress conditions, such as inflammation, lysosome dysfunction, endoplasmic reticulum stress, hypoxia or irradiation, it may lead to an increase in exosome release. It is demonstrated that tumour cells increase the release of MVs with enhanced procoagulant activity.^2^ Hypoxia induced more exosomes production in tumour cells and play important roles in tumour angiogenesis, invasion, metastasis and the immune system.^3^ It is likely that cells subjected to stress increase its communication with adjacent cells via the release of EVs.^4^

During secretion, EVs contain endogenous substances from the parent cells, including RNAs, DNAs, proteins and lipids.^5^, ^6^ Comparative study also revealed that the component varied in different pupations of EVs. While large EVs (L‐EV) and small EVs (S‐EV) (exosomes) purified from the same cells contained similar amounts of protein, DNA was more abundant in L‐EV, despite S‐EVs being more numerous.^7^ Differences in the abundance levels of the EV miRNAs could discriminate between the three EV populations, apoptotic bodies, microvesicles and exosomes.^8^ The varied cargoes of different populations of EVs may suggest their different functional roles.

## 2 BIOLOGICAL FUNCTIONS OF EVs

**Figure 1:**
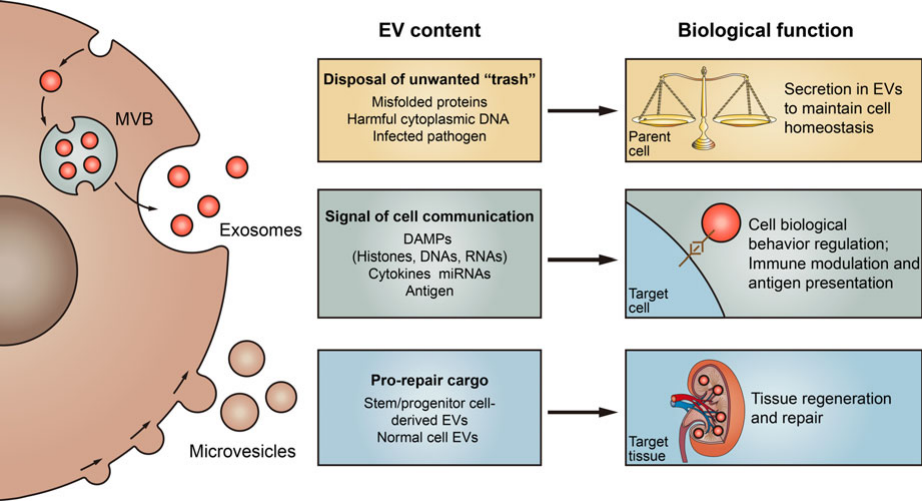
Biological function of extracellular vesicles (EVs). EVs have emerged as critical mediators in physiological processes, as well as diverse pathophysiological events. EVs is an newly identified way of maintaining cellular homeostasis by exporting harmful contents from the parent cell. Meanwhile, active molecules including DAMPs, cytokines and miRNAs are packaged into EVs that can regulate the biological behaviour of recipient cells such as proliferation and migration, or contribute to immune regulation. Besides, EVs from stem/progenitor or normal healthy cell may contain functional cargoes that are important for tissue regeneration and repair

Initially, EVs were proposed to release for getting rid of cellular waste. However, recent data revealed that EVs are an alternative way of maintaining cellular homeostasis.^9^ Importantly, these vesicles are believed to play a role in intercellular communication and have been involved in numerous cellular physiological and pathological processes (Figure 1).

### 2.1 EVs were released to maintain cell homeostasis

Multivesicular bodies can either be directed to lysosomes for degradation or transported to the plasma membrane for exosome release.^10^ Thus, exosomes were a protein quality control pathway in addition to degradation‐based approach, maintaining protein homeostasis by exporting misfolded proteins through excretion route.^11^ Autophagy is another pathway in the maintenance of protein homeostasis and the preservation of proper organelle function by selective removal of damaged organelles. Under conditions that stimulate autophagy, MVBs are directed to the autophagic pathway that consequently inhibits exosome release.^10^, ^12^ In neuronal cells, autophagy stimulation with the mTOR inhibitor rapamycin strongly inhibited exosomal prion release.^13^ When uropathogenic *E. coli* (UPEC) infect bladder epithelial cells (BECs), they are targeted by autophagy but avoid degradation because they can neutralize lysosomal pH. This change is detected by mucolipin TRP channel 3 (TRPML3) in lysosomes, initiating lysosome exocytosis and exosome‐encased bacteria.^14^ Thus, exosome biogenesis and autophagy are linked by the endolysosomal pathway to preserve intracellular protein homeostasis.

Takahashi et al reported that exosome secretion maintains cellular homeostasis by removing harmful cytoplasmic DNA from cells. The inhibition of exosome secretion results in the accumulation of nuclear DNA and consequently senescence‐like cell‐cycle arrest or apoptosis in normal human cells.^15^ However, the effect of secreted EVs packaging with DNA needs further clarification. Indeed, a recent study reports that T cell EVs that contain genomic and mitochondrial DNA can be transferred to dendritic cells (DC), inducing antiviral responses.^16^ Interestingly, mesenchymal stem cell (MSC) eliminated depolarized mitochondria by release of EVs to enhance MSCs’ cell survival.^17^

### 2.2 EVs as signalling vesicles for cell communication

As EVs were released into extracellular space, they also mediate the spreading of signals to surrounding and remote cells in addition to preserving the parent cell homeostasis. EVs may exert effects on target cells by three possible mechanisms: (a) EVs can adhere to the target cell surface via interactions between adhesion molecules and receptors present on their surfaces, leading to receptor activation of the target cell. (b) EVs could transfer their contents via membrane fusion with target cells.^18^ (c) The functional cargoes could be incorporated into target cells after endocytosis of EVs.^19^, ^20^

#### 2.2.1 EVs in immune modulation

Exosomes and microvesicles have been shown to participate in antigen presentation, immune modulation, antitumour immunity and autoimmunity. EVs can exhibit immune suppressing or activation depending on the specific circumstances and the content.^21^ EVs can modulate immune responses by transporting damage‐associated molecular patterns (DAMPs), cytokines and functional microRNAs. Alternatively, EVs could regulate immunological memory through the surface expression of antigen‐presenting MHC I and MHC II molecules.

##### EVs and DAMPs

Cells under stress or injury release EVs containing DAMPs, which can contribute to tissue inflammation. Newly identified DAMPs include extracellular heat shock proteins (eHsp72), uric acid crystals, mitochondrial DNA (mtDNA), endogenous RNAs, high mobility group box (HMGB)1 and ATP.^22^ Histones are the protein component of nucleosomes, which are the important DAMPs in tissue injury. Circulating histones contribute to inflammation by interacting with specific receptors, notably toll‐like receptor 4 (TLR4). Recent study showed histones are actively released within EVs by LPS‐activated macrophages. And histones are present on the outer surface of vesicles and can interact with TLR4.^23^ Exosome could also transfer mitochondria from airway myeloid‐derived regulatory cells to T cells, and participate in intercellular communication within the airways of human patients with asthma.^24^ Increased secretion of EV‐DNA from senescent cells may contribute to age‐related chronic inflammation.^25^

Besides, under pathological conditions, endogenous RNAs act as DAMPs for pattern recognition receptors (PRRs). RN7SL1 is an endogenous RNA that is normally shielded by RNA binding proteins. Interestingly, triggering of stromal NOTCH‐MYC by breast cancer cells results in the increase of RN7SL1 and unshielded RN7SL1 in stromal exosomes. After exosome transfer to immune cells, unshielded RN7SL1 drives an inflammatory response.^26^

##### EVs and cytokines

In addition to be secreted in soluble free format, cytokines are also imported into EVs and released into extracellular space. For instance, interleukin‐1β (IL‐1β) is a secreted protein that lacks a signal peptide and cannot be secreted in traditional pathway. Thus, IL‐1β was found to be secreted in a protected form being packaged and secreted via both exosomes and MVs.^27^, ^28^ A recent report found that a wide variety of cytokines were encapsulated into EVs as observed in different in vitro, ex vivo and in vivo systems. Importantly, EVs carrying cytokines are more stable than free cytokines and are biologically active upon interacting with sensitive cells,^29^ while free cytokines are usually unstable and have very short half‐life in plasma.^30^ EVs‐associated cytokines might be destined for signalling processes at sites distant to the local inflammatory lesion.

##### EVs and microRNA

Among EVs, exosomes are the fraction that is enriched in genetic material, mostly non‐coding RNAs. In addition to bounding to protective proteins, such as high‐density lipoprotein and argonaute protein, miRNAs were packaged into protective exosomes.^22^ Since the first study reported in 2007,^31^ increasing studies showed that exosomes carry miRNA and can transfer functionality to a recipient cell in different disease status. Adipose tissue macrophages secreted exosomes containing miRNA cargo, which can be transferred to insulin target cell types with robust effects on cellular insulin action.^32^ Another aspect of EV‐associated miRNAs that might be of importance, is that miRNA in exosomes may activate TLRs as paracrine agonists and contribute to inflammation. TLR7 and TLR8 are located in intracellular endosomes, Fabbri et al demonstrated miRNAs in cancer‐released exosomes could reach and bind TLR7 and TLR8 in a “receiving” cell.^33^

#### 2.2.2 EVs in tissue regeneration and repair

Extracellular vesicles could alter cell motility, proliferation, phenotypic change and maturation of receiving cells. For example, fibronectin is found on the outside of exosomes to support cellular adhesion and migration.^34^ Administration of EVs released from healthy cells, especially stem cells, has been shown to promoted tissue regeneration in a variety of tissue injury models. Stem/progenitor cell‐derived EVs have been demonstrated as a regenerative therapy for acceleration of wound healing in a range of clinically relevant animal models of cutaneous wounds.^33^ A number of possible mechanisms involving EV‐mediated transfer of functional molecules that trigger pro‐repair pathways in target cells have been proved.^35^ Gupta et al identified a distinct type of early apoptotic EVs with specific mitogenic activity, which are found in damaged mouse glomeruli and thus might have regenerative effects in the kidney.^36^

Interestingly, exosomes from human umbilical cord MSC inhibited STZ‐induced β‐cell apoptosis and restored the insulin secreting function of T2DM. In rat models, exosomes from hucMSC can alleviate T2DM through reversing peripheral insulin resistance and relieving β‐cell destruction.^37^ Endogenous annexin A1 (ANXA1) is released as a component of EVs derived from intestinal epithelial cells, and these ANXA1‐containing EVs activated wound repair circuits.^38^

## 3 EVs‐MEDIATED INTRANEPHRON COMMUNICATION

**Figure 2:**
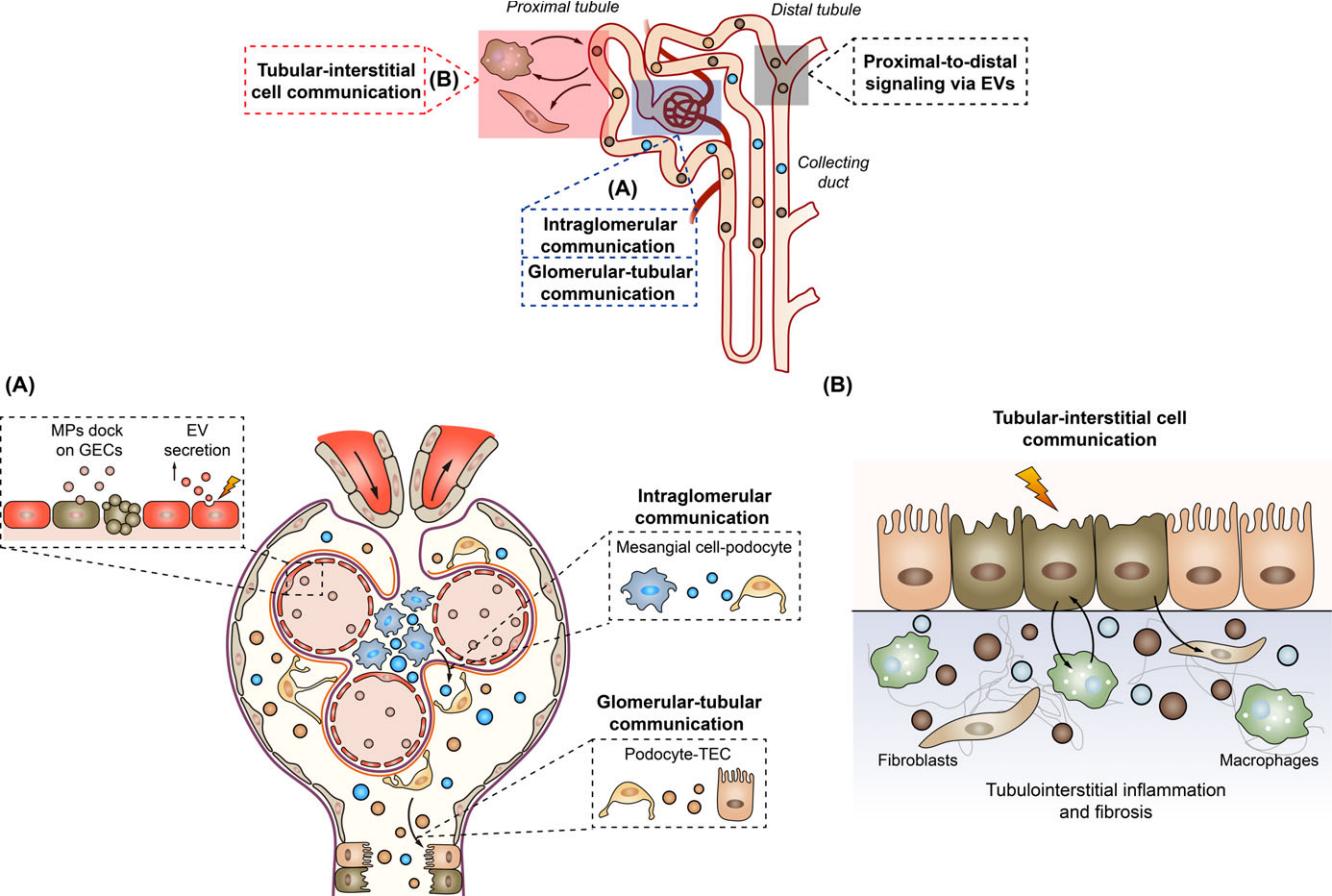
Extracellular vesicles (EVs) mediated intranephron communication. EVs are signalling vesicles for different segments of tubule, intra‐glomerular, glomerular‐tubule and tubule‐interstitial communication in physiological and pathological conditions. In glomerular, EV secretion is enhanced significantly during endothelial injury, while EVs from other cells may dock on glomerular endothelial cells (GECs) and promote endothelial dysfunction or repair. EVs also participated in the podocyte‐mesangial cell and podocyte‐tubular epithelial cells (TECs) communication (A). In tubulointerstitium, TECs communicate with interstitial macrophages and fibroblasts, promoting kidney inflammation and fibrosis (B)

It is likely that EVs secreted into the circulation and extracellular fluid have roles in renal physiology and pathophysiology through intranephron communication. The cellular crosstalk meditated by EVs especially among cells with their plasma membranes facing glomerular filtration tract or in direct contact with the vascular compartment may reasonably common in kidney. Thus, in the following part, we will focus on EV‐mediated cell communication involved in tubular epithelial cells and endothelial cells (Figure 2).

### 3.1 Tubular epithelial cell‐derived EVs and the communication routes

Tubule epithelial cells are the most populous cell type in the kidney, and carry out diverse regulatory and endocrine functions in normal kidney physiology as well as pathogenesis of kidney disease.^39^ Interestingly, recent studies indicated that external insults such as hypoxia, proteinuria or physical wounding triggered the release of EVs from tubular epithelial cells (TECs) carrying specific cargo. In condition of hypoxia, hypoxia‐inducible factor‐1 (HIF‐1) promoted exosome production in TECs.^40^ Importantly, differential expression levels of both known and unique miRNA and protein species from exosomes were found.^41^ Although the mechanism for the release of EVs from injured TECs is not clear, accumulating data have indicated that released EVs can differentially modulate the fate of neighbouring cells and consequently the severity of kidney injury.

After secretion, exosomes may have effects on the secreted cells as an endocrine factor. For example, miR‐21 was packaged into microvesicles released by TECs, which then entered recipient tubular cells, and promoted tubular phenotype transition.^42^ More likely, the secreted exosomes from TECs may travel through urinary tract or get across basement membrane and communicate with other cells as a paracrine factor.

#### 3.1.1 Proximal‐to‐distal signalling via EVs

It was demonstrated that both distal tubule and collecting duct cells could take up the EVs released by proximal tubule cells. Using culture supernatant containing exosomes from 3 CD9‐RFP and 2 CD63‐EGFP renal proximal tubule cells (RPTCs) cell lines, Gildea et al observed that all 5 distal tubule cell lines and all three collecting duct cell lines take up exosomes.^43^ AQP2 water channel is important for urinary concentration in the kidney. Interestingly, AQP2 is abundantly excreted in urinary EVs. Studies have showed that AQP2 is localized predominantly to urinary exosomes with preserved water channel activities.^44^ Importantly, the amount of AQP2 in exosomes released from collecting duct cells is physiologically regulated and exosomal AQP2 closely reflects cellular expression. Exosomes from desmopressin‐treated cells stimulated both AQP2 expression and water transport in untreated mCCDc11 cells. Thus, exosomes represent a previously unrecognized physiological mechanism for cell‐to‐cell communication in different fragments of tubules.^43^, ^45^

However, such downstream information transfer from proximal‐to‐distal has not been demonstrated in in vivo study. van Balkom et al speculated that Tamm‐Horsfall protein (uromodulin) may limit exosomal fusion in downstream nephron segments, because urinary exosomes are usually shrouded by polymeric fibres formed from Tamm‐Horsfall protein, which would prevent them from getting contact with surfaces of target cells.^19^, ^46^ The effect of Tamm‐Horsfall protein on urinary exosome communication with downstream tubule segment needs further investigation.

#### 3.1.2 Tubular‐interstitial cell communication

Recent evidence demonstrates that enzymatically active proteases and glycosidases are present on the surface of some exosomes, which can degrade the extracellular matrix and facilitate cell adhesion and invasion. Enzymatic functions of exosomes have implications in the progression of cancer, inflammation and Alzheimer's disease.^47^, ^48^ Thus, it is reasonably to speculate that tubular exosomes could get across basement membrane to communicate with interstitial cells especially when the permeability of the filtration barrier increased during kidney injury.

Indeed, previous study showed that TECs communicated with interstitial macrophages during kidney injury via soluble molecules. Wang et al reported that expression of soluble epoxide hydrolase in renal TECs regulates macrophage infiltration and polarization in IgA nephropathy.^49^ Interestingly, EVs pass from injured TECs to interstitial space via damaged basement membrane also contributed to macrophage activation. Upon exposure to proteinuria, TECs produced increasing exosomes loading with CCL2 mRNA which could be transferred to macrophages and promoted macrophage activation. It may constitute a critical mechanism of albumin‐induced tubulointerstitial inflammation.^50^ Interestingly, in tumour microenvironment, exosome‐mimetic nanovesicles derived from M1 macrophages could induce polarization of M2 macrophages to M1 macrophages in vitro and in vivo. Thus, exosome may represent a novel mediator for inducing macrophage polarization.^51^

Moreover, TEC exosomes also participated in the development of renal fibrosis through communication with interstitial fibroblast. Borges FT et al reported that exosomes released by injured epithelial cells promote fibroblast activation that is dependent on exosomes delivering of TGF‐β1 mRNA. The study indicated the potential utility of exosome‐targeted therapies to control tissue fibrosis.^52^ As EV‐associated MMPs can contribute to degradation of extracellular matrix surrounding cells, and sometimes stimulate critical signalling pathways,^47^, ^48^, ^53^ whether EV‐associated MMPs participated in the development of renal fibrosis is an interesting question that deserves further investigation.

In addition to secretion of EVs to spread signals, TECs also accept information from other cells via EVs. When macrophages were incubated with calcium oxalate (CaOx) crystals, exosomes were secreted to enhance IL‐8 production in renal tubular cells.^54^ Microparticles (MPs) released by activated endothelial cells up‐regulate HIF‐1/HIF‐2 and increase the production of HIF‐/VEGF‐A in human proximal tubular cells. Thus, the presence of endothelial MPs in the urinary space may influence the outcome of renal diseases through communication with TECs.^55^

#### 3.1.3 Effects of TEC‐EVs on kidney injury and repair

Zhang et al reported that in cell culture study, exosomes from hypoxic renal proximal tubular cells (RPTCs) had inhibitory effects on apoptosis of RPTCs following ATP depletion.^40^ Intravenous administration of exosomes from normal human kidney tubular cells prevented damage after hypoxic AKI.^56^, ^57^ In contrary to the protective effect, other study found that exosomes from injured TECs accelerated kidney injury through activating macrophage infiltration and activation in tubulointerstitium.^50^ Similarly, scratch wounding in TECs induced a significant increase of exosome production, and the secreted exosomes could inhibit wound healing.^58^  Thus, diverse biological effects of exosomes from TECs have been described in different models which might depend on the conditions and functional state of the parent cells.

### 3.2 EVs mediated intra‐glomerular and glomerular‐tubular communication

Study showed that interaction between glomerular mesangial cells and podocytes via exosomes might affect function of glomerulus in diabetic nephropathy condition. Transwell system showed that the exosomes released by glomerular mesangial cells under high glucose condition were involved in podocyte injury. High glucose promoted TGFβ1 loading into exosomes in glomerular mesangial cells, while berberine can reduce the level of TGFβ1 in exosomes and can protect damage of podocytes by reducing apoptosis and increasing adhesion.^50^

Podocyte exosomes were secreted into urine and might pass through the renal tubule and transmit information to tubular epithelial cells.^59^ Given its location adjacent to the glomerulus, the proximal tubule represents a possible site of interaction for podocyte EVs. It has been demonstrated in in vitro study, that podocyte MPs did communicate with proximal tubule epithelial cells (PTECs) and induced the cell fibrotic responses. MPs were isolated from the media of differentiated, untreated human podocytes (hPODs) and administered to cultured PTECs. Treatment with podocyte MPs promoted proximal tubule fibrotic signalling via p38 MAPK and CD36.^60^ However, in this study, MPs were from the normal podocyte, it is still unclear what are the effects of MPs from injured podocytes on tubular epithelial cells. Moreover, the difference for normal and injured PTECs in internalizing podocyte MPs deserves further investigation.

### 3.3 EVs and endothelial dysfunction and repair

Endothelial cell injury is central to the pathophysiology of acute and chronic kidney injury due to oxygen depletion, reactive oxygen species generation and hyperperfusion.^61^, ^62^ EVs release was enhanced significantly during endothelial injury.  In acute vasculitis, higher level of circulating endothelial microvesicles was observed. Importantly, kinin B1 receptor‐positive microvesicles induced neutrophil chemotaxis and contribute to inflammatory process.^63^ On the other hand, the same group demonstrated that leukocyte‐derived microvesicles bearing B1‐kinin receptors are enriched in the plasma of vasculitis patients and dock on endothelial cells in the glomerulus. Thus, leukocyte microvesicles transfer functional receptors to endothelial cell and promote kinin‐associated inflammation.^64^ Moreover, endothelial EVs levels, especially endothelial microparticles (EMPs) might also be useful biomarkers for chronic kidney disease (CKD) population and haemodialysis patients, which were associated with greater mortality.^65^, ^66^

Recent study revealed that EVs‐derived bioactive molecules from different cell sources promote endothelial regeneration. Following treatment of injured endothelial cells with renal artery‐derived vascular progenitor cells (RAPC)‐derived exosomes, endothelial migration improved and stimulates a reparative phenotype.^67^ MPs derived from kidney‐derived mesenchymal stem cells (KMSCs) have been reported to ameliorate rarefaction of peritubular capillaries (PTC) in ischaemic kidneys via delivery of proangiogenic effectors. Besides, KMSC‐derived MPs significantly inhibited endothelial‐to‐mesenchymal transition (EndoMT) of PTC endothelial cells and improved PTC rarefaction as well as tubulointerstitial fibrosis in UUO kidneys.^68^ Exosomes derived from endothelial colony‐forming cells (ECFCs) were also shown to alleviate apoptosis of the endothelial cells and stimulate capillary endothelium repair.^69^

However, in other situations, EVs were harmful to endothelial function. Serum exosomes (SExos) from diabetic *db/db* mice (*db/db* SExos) were taken up by aortic endothelial cells, which severely impaired endothelial function in nondiabetic *db/m^+^* mice. Comparative proteomics analysis revealed arginase 1 protein played essential role in *db/db* SExos‐induced endothelial dysfunction.^70^

## 4 EV AS BIOMARKERS IN KIDNEY DISEASE

Extracellular vesicles are found in almost all biofluids and EVs' cargo changed with disease states, positioning EVs as potential sources for the discovery of novel biomarkers of disease. RNAs (mRNA and miRNA) and proteins from kidney resident or infiltrating cells can be loaded into urinary EVs and thus protected from degradation. Urinary EVs might be useful sources of novel biomarker for renal disease.

For example, urinary EV UMOD mRNA levels are progressively elevated from T2DM to DKD groups and correlate with eGFR and ACR levels.^71^ We have recently isolated exosome released from podocyte in the urine, the vesicle structure was positive for the markers of both exosome and podocyte, CD9, AQP2 and nephrin. Further study showed that CD2AP mRNA from exosome was correlated with both kidney function and severity of fibrosis.^72^ Importantly, urinary exosomes and exosomal CCL2 mRNA are promising biomarkers reflecting active renal histologic injury and predicting renal function deterioration in IgAN.^73^

Moreover, the stability and enrichment of miRNA in exosome make it a promising candidate biomarker for kidney disease. Exosome miRNA was stable despite repeated frozen‐thaw cycles and long‐term storage.^74^ Interestingly, miRNAs were extremely enriched in the urinary exosome subpopulation, but not MVs in hypertensive patients. Low exosomal miR‐146a was associated with the presence of albuminuria.^75^ MiR‐29c from urinary exosome was significantly reduced in CKD patients and inversely correlated with renal fibrosis scores.^76^ Besides, the diagnostic potential of exosomal miRNA has also been demonstrated in autosomal dominant polycystic kidney disease (ADPKD), streptozotocin (STZ)‐induced diabetic nephropathy animal models, patients with minimal change disease (MCD) and focal segmental glomerulosclerosis (FSGS).^77^, ^78^, ^79^ However, urinary exosomal biomarkers in different types of kidney disease warrant further validation studies.

## 5 CHALLENGES AND FUTURE DIRECTIONS OF EVs STUDY IN KIDNEY DISEASE

Despite all the promising data regarding EVs’ function in kidney disease, more researches are still needed to extend these findings. First of all, as it is technically challenging to obtain a totally pure EV fraction free from non‐vesicular components, it is essential to establish comprehensive studies when EV function was studied. Recently, International Society for Extracellular Vesicles (ISEV) reported the minimal experimental requirements that should be used to attribute any specific biological cargo or functions to EVs.^80^ More importantly, nearly all results describing the exosome‐mediated cellular crosstalk so far are based on the experimental data in vitro or partly in rodent models. The major impediment to mechanistic studies is to clarity how EVs are formed and how to track their origin and destination in vivo.^81^ Identifying the tissues or specific cell of origin for circulating EVs may be especially important in understanding their relevance to disease.^81^ Although it is possible to isolate tubule exosomes through glomerular and tubule separation, it is still difficult to identify other cell‐specific origin of EVs from kidney.

For urinary EV biomarker study, more efforts are necessary to standardize the EVs isolation methods and the nomenclature of EVs, which might help to get more comparable and repeatable data.^81^, ^82^, ^83^ Importantly, the low purity of EVs obtained from different isolation methods is common. A recent study report that ultracentrifugation followed by size exclusion chromatography (UC‐SEC) yielded the most homogenous population of exosomes and less non‐vesicle co‐precipitated proteins from urine.^84^ The optimal method for EV purification is important especially for urine samples from proteinuric nephropathy patients.^85^ Besides, large cohort of validation study is necessary for biomarker development and clinic translation. Meanwhile, to determine the cell origin of urinary exosomes and the relevance of candidate biomarker to the development of kidney disease are the critical issues that deserve in‐depth consideration. Cell‐specific exosome isolation might rely on the specific cell and exosome markers as well as exosome‐bound bead and flow cytometry analysis. However, the efficiency and practicability need further investigation.

In summary, EVs mediated cellular crosstalk in between kidney resident cells and infiltrating cells, promoting the spread of injury signal or contributing to kidney repair. EVs released from different nephron districts may participate in intranephron communication in physiological and pathological conditions. EV's cargo, especially urinary exosomes may represent promising biomarker for kidney disease, reflecting histological changes and kidney function.

## CONFLICT OF INTEREST

The authors declare that they have no competing interests.
